# Risk factors for complications after pharyngolaryngectomy with total esophagectomy

**DOI:** 10.1007/s10388-016-0533-9

**Published:** 2016-03-31

**Authors:** Eisuke Booka, Yasuhiro Tsubosa, Masahiro Niihara, Wataru Takagi, Katsushi Takebayashi, Ayako Shimada, Takashi Kitani, Masato Nagaoka, Atsushi Imai, Tomoyuki Kamijo, Yoshiyuki Iida, Tetsuro Onitsuka, Masahiro Nakagawa, Hiroya Takeuchi, Yuko Kitagawa

**Affiliations:** 1 Division of Esophageal Surgery, Shizuoka Cancer Center Hospital, 1007 Shimonagakubo, Nagaizumi-cho, Sunto-gun, Shizuoka, 411-8777 Japan; 2 Division of Head and Neck Surgery, Shizuoka Cancer Center Hospital, 1007 Shimonagakubo, Nagaizumi-cho, Sunto-gun, Shizuoka, 411-8777 Japan; 3 Division of Plastic and Reconstructive Surgery, Shizuoka Cancer Center Hospital, 1007 Shimonagakubo, Nagaizumi-cho, Sunto-gun, Shizuoka, 411-8777 Japan; 4 Department of Surgery, Keio University School of Medicine, 35 Shinanomachi, Shinjuku-ku, Tokyo 160-8582 Japan

**Keywords:** Pharyngolaryngectomy, Total esophagectomy, Tracheal necrosis, Esophageal cancer, Hypopharyngeal cancer

## Abstract

**Background:**

Pharyngolaryngectomy with total esophagectomy (PLTE) is an effective surgical treatment for synchronous or metachronous hypopharyngeal or laryngeal cancer and thoracic esophageal cancer, although it is more invasive than esophagectomy and total pharyngolaryngectomy. The aim of this study was to identify risk factors for complications after PLTE.

**Methods:**

From November 2002 to December 2014, a total of 8 patients underwent PLTE at the Shizuoka Cancer Center Hospital, Shizuoka, Japan. We investigated the clinicopathological characteristics, surgical procedures, and postoperative complications of these patients.

**Results:**

Of the 8 patients, 5 underwent one-stage PLTE and 3 underwent staged PLTE. There was no mortality in this study. Two cases of tracheal necrosis, two of anastomotic leakage, and one of ileus were observed as postoperative complications. Two patients who underwent one-stage PLTE with standard mediastinal lymph node dissection developed tracheal necrosis and severe anastomotic leakage.

**Conclusion:**

One-stage PLTE and standard mediastinal lymph node dissection were identified as the risk factors for severe postoperative complications. Staged PLTE or transhiatal esophagectomy should be considered when PLTE is performed and standard mediastinal lymph node dissection should be avoided when one-stage PLTE is performed with transthoracic esophagectomy.

## Introduction

Esophageal cancer is the sixth leading cause of cancer-related mortality worldwide because of its high malignant potential and poor prognosis [1]. The postoperative 5-year survival rate in patients with American Joint Committee on Cancer stage I esophageal cancer is approximately 90 %; it decreases to 45 % in patients with stage II disease, 20 % in those with stage III disease, and 10 % in those with stage IV disease [2]. Although the efficacy of chemoradiotherapy for esophageal cancer has been reported [3], esophagectomy remains the most viable treatment option for esophageal cancer. However, esophagectomy is a highly invasive procedure associated with several serious postoperative complications such as pneumonia, anastomotic leakage, and recurrent laryngeal nerve paralysis, which may result in multiorgan failure [4]. Another clinical problem associated with esophageal cancer is its frequent association with synchronous or metachronous gastric or head and neck cancer [5]. Similar to the difficulty regarding the treatment for gastric tube cancer after esophagectomy previously reported by us [5], it is complicated to treat esophageal cancer with synchronous or metachronous head and neck cancer. Pharyngolaryngectomy with total esophagectomy (PLTE) is an effective surgical treatment for synchronous or metachronous hypopharyngeal or laryngeal and thoracic esophageal cancers, although PLTE is more invasive than esophagectomy and total pharyngolaryngectomy (TPL) [6–9].

The fatal complications associated with PLTE are tracheal and gastric tube necrosis caused by insufficient blood flow [10]. PLTE is also indicated for cervicothoracic and cervical esophageal cancers with mediastinal lymph node metastasis [8]. To date, although a few earlier studies have reported the efficacy of PLTE for synchronous or metachronous pharyngeal and thoracic esophageal cancers [6–8], there has been no study investigating the differences between one-stage and staged PLTE. Therefore, this study is the first to draw a comparison between one-stage and staged PLTE. We hypothesized that the chosen surgical procedure for PLTE likely impacts the potential development of postoperative complications. Hence, the aim of this study was to identify potential risk factors for complications after PLTE.

## Patients and methods

### Patients

From November 2002 to December 2014, a total of 375 patients underwent esophagectomy and 140 patients underwent TPL at Shizuoka Cancer Center Hospital, Shizuoka, Japan. In this study, 8 patients who underwent PLTE were retrospectively analyzed. Of these patients, 5 underwent one-stage PLTE and 3 underwent staged PLTE for metachronous pharyngeal and thoracic esophageal cancers. Of the 5 patients who underwent one-stage PLTE, 2 underwent PLTE based on the indication of cervicothoracic esophageal cancer, 2 underwent PLTE based on the indication of synchronous pharyngeal and thoracic esophageal cancers, and 1 underwent PLTE based on the indication of synchronous cervical and thoracic esophageal cancers. Of the 3 patients who underwent staged PLTE for metachronous pharyngeal and thoracic esophageal cancers, 2 underwent TPL followed by esophagectomy and 1 underwent esophagectomy followed by TPL. Clinical staging of esophageal and pharyngeal cancers was categorized according to the International Union Against Cancer (UICC) 7th edition tumor–node–metastasis (TNM) classification [11].

### Preoperative treatment

Of the 8 patients who underwent PLTE, 7 received preoperative treatment of definitive chemoradiotherapy (dCRT) (5 patients) or neoadjuvant chemotherapy (2 patients). Definitive chemoradiotherapy comprised the concurrent administration of approximately 60 Gy radiation with 5-fluorouracil and cisplatin. Salvage surgery was indicated for residual or recurrent lesions after dCRT. Neoadjuvant chemotherapy comprised 5-fluorouracil and cisplatin.

### Surgical procedure

Esophagectomy was performed through right thoracotomy, video-assisted thoracic surgery (VATS), or transhiatal procedures by esophageal surgeons. Esophagectomy through right thoracotomy or VATS included standard mediastinal lymph node dissection. However, the dissection was avoided when salvage surgery was performed considering the high risk of complications associated with the surgery. TPL was performed by esophageal or head and neck surgeons according to the tumor location. In this study, the reconstructed organ was the stomach in all cases. Pharyngogastric anastomosis was performed by esophageal surgeons when the whole stomach or pulled-up gastric tube could reach the hypopharynx. When the gastric tube had been pulled up but could not reach the hypopharynx, free jejunal transfer (FJT) with microvascular anastomosis was performed to repair the cervical defect between the hypopharynx and the oral side of the gastric tube by plastic and reconstructive surgeons. Two anastomoses were required in this case: pharyngojejunal and jejunal gastric. Postoperative complications were categorized using the Clavien–Dindo (CD) classification [12, 13].

## Results

### Patient and clinicopathological characteristics

The clinicopathological characteristics of patients who underwent one-stage PLTE and staged PLTE are summarized in Tables 1 and 2, respectively. The median age at the time of PLTE and first staged PLTE was 63.5 years (range 43–69 years). The study cohort of 8 patients comprised 6 males and 2 females. Standard mediastinal lymph node dissection was performed in all patients of staged PLTE (patients 6, 7, and 8) and 2 patients of one-stage PLTE (patients 4 and 5), whereas standard mediastinal lymph node dissection was avoided in all patients of salvage surgery (patients 1, 2, and 3).

**Table 1:** Clinicopathological characteristics of patients who underwent one-stage PLTE

Patient	Age (years)	Sex	Cancer Site	cTNM (UICC 7th)	Preoperative treatment	Method of esophagectomy	Standard mediastinal lymph node dissection	Reconstructed method	Reconstructed route
1	64	Male	Ce	T4bN1M0	dCRT (2 months after radiation)	Transhiatal	No	Whole stomach	Posterior mediastinal
2	69	Male	UtCe	T2N1M0	dCRT (2 months after radiation)	Transhiatal	No	Gastric tube + FJT	Posterior mediastinal
3	66	Male	Ph	T2N0M0	dCRT (52 months after radiation)	Transthoracic	No	Gastric tube + FJT	Posterior mediastinal
4	57	Female	Mt Ce	T1aN1M0 T3N0M0	NeoFP	Transthoracic	Yes	Gastric tube + FJT	Posterior mediastinal
5	63	Male	Mt Ph Mt	T1N0M0 T4aN2bM0 T1bN1M0		VATS	Yes	Gastric tube + FJT	Posterior mediastinal

*PLTE* Pharyngolaryngectomy with total esophagectomy, *cTNM* clinical tumor–node–metastasis, *UICC* international union against cancer, *Ph* pharynx, *Ce* cervical esophagus, *Ut* upper thoracic esophagus, *Mt* middle thoracic esophagus, *dCRT* definitive chemoradiotherapy, *NeoFP* neoadjuvant chemotherapy comprised 5-fluorouracil and cisplatin, *VATS* video-assisted thoracic surgery, *FJT* free jejunal transfer

**Table 2:** Clinicopathological characteristics of patients who underwent staged PLTE

Patient	Age (years)	Sex	First site of cancer	cTNM (UICC 7th)	Preoperative treatment	First operation	Duration (months)	Second site of cancer	cTNM (UICC 7th)	Preoperative treatment	Second operation
6	43	Female	Ph	T4aN2bM0		TPL + FJT	25	Lt	T1bN1M0	NeoFP	TTE + posterior mediastinal gastric tube
7	64	Male	Ce	T4bN0M0	dCRT (36 months after radiation)	TPL + FJT	1	Mt	T1bN0M0		TTE + posterior mediastinal gastric tube
8	57	Male	MtUtLt	T4bN1M0	dCRT (3 months after radiation)	TTE + retrosternal gastric tube	23	Ph	T3N2cM0		TPL + FJT

*PLTE* Pharyngolaryngectomy with total esophagectomy, *cTNM* clinical tumor–node–metastasis, *UICC* international union against cancer, *Duration* duration between operations (months), *Ph* pharynx, *Ce* cervical esophagus, *Ut* upper thoracic esophagus, *Mt* middle thoracic esophagus, *Lt* lower thoracic esophagus, *dCRT* definitive chemoradiotherapy, *NeoFP* neoadjuvant chemotherapy comprised 5-fluorouracil and cisplatin, *TPL* total pharyngolaryngectomy, *FJT* free jejunal transfer, *TTE* transthoracic esophagectomy

Patients 1 and 2 underwent dCRT and salvage PLTE for residual lesions. Patient 3 underwent dCRT for pharyngeal cancer, which resulted in a complete response. However, pharyngeal cancer recurrence after dCRT and synchronous esophageal cancer was observed in 2 lesions, which were subsequently treated by PLTE. Patients 4 and 5 underwent PLTE for synchronous double cancers.

Patient 6 underwent TPL with FJT as the first surgery. However, 25 months later, this patient underwent esophagectomy with posterior mediastinal gastric tube reconstruction. Patient 7 underwent salvage TPL with FJT for severe stenosis after dCRT followed 1 month later by esophagectomy with posterior mediastinal gastric tube reconstruction as the second operation. Patient 8 underwent salvage esophagectomy with retrosternal gastric tube reconstruction for residual lesions after dCRT. Twenty-three months after salvage esophagectomy, this patient underwent TPL with FJT for metachronous pharyngeal cancer. A second operation was performed for palliative care, and a portion of the cervical esophagus was preserved with minimum invasiveness.

### Clinical outcome after PLTE

The clinical outcomes after PLTE for all 8 patients are shown in Table 3. For the patients who underwent staged PLTE (patients 6, 7, and 8), there were no postoperative complications from the first operation. The perioperative and postoperative outcomes of the second operation are shown in Table 3.

**Table 3:** Clinical outcome after PLTE for all 8 patients

Patient	Surgical duration (min)	Blood loss (ml)	Complications (CD classification)	Hospital stay (day)	Outcome
1	443	1430		30	Dead at 14 months (lung metastasis)
2	730	1002	Anastomotic leakage (II)	27	Alive at 38 months
3	415	580		25	Alive at 37 months
4	536	183	Tracheal necrosis (IIIa)	25	Alive at 23 months
5	724	384	Anastomotic leakage (IIIb), tracheal necrosis (IIIa)	99	Alive at 12 months
6	414	631		18	Alive at 89 months
7	587	735	Ileus (IIIb)	45	Dead at 53 months (pneumonia)
8	702	179		32	Alive at 7 months (lymph node metastasis)

*PLTE* Pharyngolaryngectomy with total esophagectomy, *CD* Clavien–Dindo

The mean operation time was 569 min (range 443–730 min) and the median blood loss was 640 ml (range 179–1430 ml). The median hospital stay was 28.5 days (range 18–99 days). There was no instance of mortality in this study. Two cases of tracheal necrosis (patients 4 and 5), 2 of anastomotic leakage (patients 2 and 5), and 1 of ileus (patient 7) were observed as postoperative complications.

**Fig. 1:**
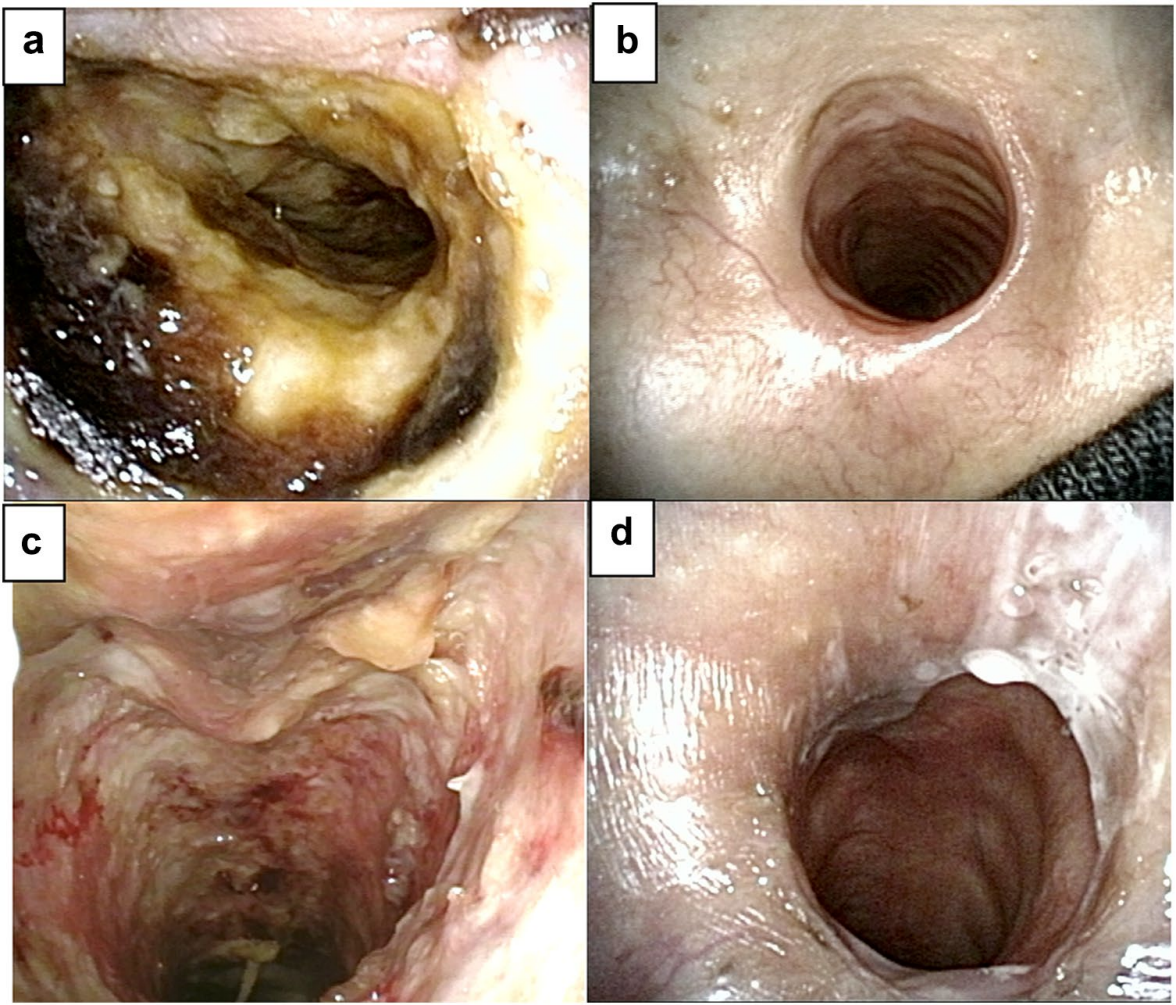
**a**, **b** Tracheal necrosis on postoperative day (POD) 9 (**a**) and improvement on POD 164 (**b**) in case 4. **c**, **d** Tracheal necrosis on postoperative day (POD) 13 (**c**) and improvement on POD 113 (**d**) in case 5

The occurrence of tracheal necrosis is shown in Fig. 1 (patients 4 and 5). In case of patient 4, tracheal necrosis was observed on postoperative day (POD) 9 (Fig. 1a) and a tracheostomy tube was inserted against tracheal stenosis on POD 79. Tracheal necrosis improved on POD 164 (Fig. 1b). In case of patient 5, tracheal necrosis was observed on POD 13 (Fig. 1c) and a tracheostomy tube was inserted against tracheal stenosis on POD 16. The membranous portion of the trachea was melted on POD 26 and fistula formation was observed on POD 40. Fistula closure was performed on POD 77 and tracheal necrosis improved on POD 113 (Fig. 1d).

Patient 1 died because of lung metastasis at 14 months and patient 7 died because of pneumonia that were not related to esophageal cancer at 53 months. The remaining 6 patients are alive, including 1 who experienced lymph node recurrence (patient 8) and 5 who experienced no recurrence.

## Discussion

PLTE is an effective surgical treatment for synchronous or metachronous hypopharyngeal or laryngeal and thoracic esophageal cancers. However, PLTE is more invasive than esophagectomy or TPL, and it is important to prevent postoperative complications and consider indications for this invasive procedure [6–8, 14].

At our institution, the eligibility criteria for PLTE were not clearly defined. However, we decided the criteria after careful consideration of all the factors that would comprehensively affect patient life. As a result, the average age of participants in this study cohort was less than usual cohort of esophageal cancer [15].

In this study, tracheal necrosis developed in patients 4 and 5 and severe anastomotic leakage developed in patient 5, and the severe anastomotic leakage was believed to stem from tracheal necrosis. Subsequently, these patients underwent transthoracic or thoracoscopic esophagectomy with standard mediastinal lymph node dissection, and it was possible that insufficient tracheal blood flow developed in response to standard mediastinal lymph node dissection [16]. Tracheal necrosis is considered to develop in response to insufficient tracheal blood flow [10, 16]. Patient 3 underwent transthoracic esophagectomy; however, standard mediastinal lymph node dissection was avoided considering the high risk associated with salvage surgery [17]; therefore, tracheal blood flow was preserved. For the 2 patients (patients 1 and 2) who underwent transhiatal esophagectomy, tracheal blood flow was preserved because mediastinal lymph node dissection was not performed. For the 3 patients (patients 6, 7, and 8) who underwent staged PLTE, transthoracic esophagectomy with standard mediastinal lymph node dissection was performed. However, tracheal blood flow bypass was thought to have been created in response. One-stage PLTE and standard mediastinal lymph node dissection were identified as risk factors of severe postoperative complications, particularly tracheal necrosis.

Staged PLTE was performed for metachronous cancers but not planed as the first operation in this study. In the second operation, when the staged PLTE required efforts and cervical esophagus could not be resected in cases such as patient 8, staged PLTE may have contributed to the occurrence of tracheal blood flow bypass and was considered to be an effective procedure. Moreover, some reports have claimed that staged PLTE is safe and effective for high-risk patients [7, 8]. In this study, the shortest duration between the first and second procedures was 1 month, and tracheal blood flow bypass can occur within this period. Nonetheless, our experience indicates that staged PLTE could be effective and safe for both metachronous and synchronous cancers.

Definitive CRT tends to be the initial treatment for synchronous double head and neck and thoracic esophageal cancers [7]. However, some salvage treatment is required in cases with either residual or recurrent disease after dCRT [7]. It is considered that salvage surgery after dCRT is a high-risk factor for severe postoperative complications [9, 17]. In this study, 5 patients (62.5 %) underwent salvage PLTE after dCRT, which resulted in no instance of tracheal necrosis or severe anastomotic leakage. Therefore, we propose that salvage surgery can be safely performed when staged PLTE or transhiatal esophagectomy is selected.

In this study, those 2 patients who developed tracheal necrosis developed tracheal stenosis when tracheal necrosis improved. In these 2 patients, insertion of a tracheostomy tube was effective to improve tracheal stenosis. In cases in which tracheal stenosis cannot be avoided when tracheal necrosis improves, a tracheostomy tube should be inserted to treat tracheal stenosis [18].

In conclusion, one-stage PLTE and standard mediastinal lymph node dissection were identified as risk factors for severe postoperative complications. Staged PLTE or transhiatal esophagectomy should be considered when PLTE is performed and standard mediastinal lymph node dissection should be avoided when one-stage PLTE is performed with transthoracic esophagectomy.
